# Protective Association of APOC1/rs4420638 with Risk of Obesity: A case-control Study in Portuguese Children

**DOI:** 10.1007/s10528-023-10427-4

**Published:** 2023-06-16

**Authors:** Licínio Manco, David Albuquerque, Daniela Rodrigues, Aristides M. Machado-Rodrigues, Cristina Padez

**Affiliations:** 1 Research Centre for Anthropology and Health (CIAS), University of Coimbra, Coimbra, 3000 Portugal; 2 Department of Life Sciences, University of Coimbra, Coimbra, Portugal; 3 Faculty of Sport Sciences and Physical Education, University of Coimbra, Coimbra, Portugal

**Keywords:** APOC1 gene, rs4420638, APOE gene, Childhood obesity, Portugal

## Abstract

The association of the rs4420638 polymorphism, near the APOC1 gene, was examined with the risk of obesity among Portuguese children. A sample of 446 Portuguese individuals (231 boys and 215 girls) of European descent, aged 3.2 to 13.7 years old (mean age: 7.98 years), were selected to conduct a case-control study. Body mass index (BMI), BMI Z-scores, and waist circumference were calculated. Genotyping was performed by real time PCR using a pre-designed TaqMan probe. Logistic regression and the nonparametric Mann-Whitney test were used to test the associations. The association results revealed a significant protective effect from the minor G-allele of SNP rs4420638 against obesity, with an odds ratio (OR) of 0.619 (95% CI 0.421–0.913; *p* = 0.0155) in the additive model, and OR of 0.587 (95% CI 0.383-0.9; *p* = 0.0145) in the dominant model. Moreover, comparing genotype groups (AA vs. AG + GG), significantly lower values (*p* < 0.05) for the anthropometric traits weight, height, BMI, BMI Z-score and waist circumference, were observed in the carriers of allele G. The present study provides further evidence for the *APOE*/*APOC1* candidate-region association with the risk of obesity. This was the first study to describe the protective association of the rs4420638 minor G-allele against obesity in childhood exclusively.

**Supplementary Information:**

The online version contains supplementary material available at 10.1007/s10528-023-10427-4.

## Introduction

The prevalence of overweight and obesity in children and adolescents has increased enormously in the last few decades, both in girls and boys (Ng et al. 2014). This epidemic proportion reached by obesity in the pediatric population suggests the importance of environmental factors, mostly the consumption of high-calorie foods and a sedentary lifestyle (Vilchis-Gil et al. 2015). However, it is well established that childhood obesity results from a complex interplay between genetic and these non-genetic environmental factors (Mason et al. 2020; Loos and Yeo 2022). Furthermore, some studies have elucidated epigenetic modifications with a role in weight gain (Alfano et al. 2022).

The SNP rs4420638 (NM_001645.3, c.*459A > G) located approximately 340 bp from the 3’ end of the apolipoprotein C-I (*APOC1*) gene, in the chromosomal region 19q13.32, tags a linkage disequilibrium (LD) block involving the *TOMM40*, *APOE*, and *APOC1* genes (Suchindran et al. 2010; Beekman et al. 2013), and has been implicated in dyslipidemia by numerous genetic association studies [GWAS Catalog EMBL-EBI (http://www.ebi.ac.uk/gwas; accessed on January 24, 2023)] (Sollis et al. 2023). The rs4420638 A > G polymorphism was significantly related with activity of lipoprotein-associated phospholipase A2 (Lp-PLA2) and serum levels of total cholesterol (TC), triglyceride (TG), low-density-lipoprotein (LDL) cholesterol or high-density lipoprotein (HDL) cholesterol (Kathiresan et al. 2008; Drenos et al. 2009; Suchindran et al. 2010; Liu et al. 2011; Grallert et al. 2012; Breitling et al. 2015; Zhang et al. 2018; Wang et al. 2022). Furthermore, rs4420638 was associated with risks of metabolic syndrome, type 2 diabetes, and coronary heart disease (Zhang et al. 2018). However, the mechanism by which the rs4420638 A > G variant influences lipid metabolism and risk of diseases or whether these associations are related with the LD observed with *APOE* polymorphisms remain to be clarified (Zhang et al. 2018).

Additionally, while a few studies have identified rs4420638 as an obesity-linked genetic variant (Winkler et al. 2015; Lotta et al. 2018; Boulenouar et al. 2019), some did not reveal this association (Wang et al. 2022). Moreover, to our knowledge, there is no studies on the relationship between obesity and the rs4420638 variant in childhood exclusively. Considering these conflicting results for rs4420638 as an obesity-associated variant, and the total lack of studies exploring the association of this genetic variant with the risk of obesity in the Portuguese population, we conducted the present case-control study in a sample of Portuguese children.

## Materials and Methods

### Study Population

Participants were randomly recruited from several pre- and primary public schools in the Portuguese Midlands between 2013 and 2018 in two independent studies carried out (Albuquerque et al. 2013; Manco et al. 2019). Both projects were approved by the *Direcção Geral de Inovação e de Desenvolvimento Curricular* of the Ministry of Education, and they were conducted anonymously, and according to the guidelines laid down in the Declaration of Helsinki. Written informed consent was obtained from all children’s parents or tutors. From a total of 1170 Portuguese children, 446 of them (231 boys and 215 girls) from European descent, aged 3.2 to 13.7 years-old (mean 7.98 years), were selected to conduct the present case-control study.

The anthropometric measurements of height, weight and waist circumference were taken by trained researchers according to established protocols, as described elsewhere (Albuquerque et al. 2013; Manco et al. 2019). Body mass index (BMI) was calculated. Overweight and obesity were classified using age and sex specific BMI cut-offs adopted by the International Obesity Task Force (IOTF) (Cole et al. 2000). A child was considered with overweight or obesity when the BMI was above the 85th percentile (i.e., overweight if 95th > BMI ≥ 85th percentile, corresponding to Z-scores between + 1 and < + 2 SD; obesity if BMI ≥ 95th percentile, corresponding to Z-scores ≥ + 2 SD) (de Onis et al. 2007). Within the 446 participants, 234 were classified has having normal-weight, and 212 had overweight or obesity.

### Genotyping

Buccal swabs were collected from each participant and the genomic DNA was extracted using the FavorPrep™ Genomic DNA Mini Kit (Favorgen® Biotech Corp, Taiwan). Polymorphism rs4420638 was genotyped by real-time polymerase chain reaction (qPCR) using the pre-designed TaqMan SNP® Genotyping assay C__26682080_10 (Applied Biosystems, Foster City, CA) in a CFX96 Touch Real-Time PCR Detection System (Bio-Rad Laboratories, CA). The PCR amplification was carried out in 10 µl of a total reaction volume containing 1 µl (~ 10 ng) of DNA, 0.25 µl (20x) of TaqMan SNP assay in 1x of SsoFast™ Probes Supermix (Bio-Rad, Hercules, CA, USA). PCR conditions were an initial denature step at 95 °C for 10 min, followed by 40 cycles of 1 min at 62 °C and 15 s at 95 °C. To assess genotyping reproducibility, a 10% random selection of samples were re-genotyped.

### Statistical Analysis

Genotype and allele frequencies were estimated by direct counting and the Hardy-Weinberg equilibrium (HWE) probability value was achieved using an exact test. Differences in the genotype distribution between cases and controls were tested using logistic regression, under the additive (AA vs. AG vs. GG) and dominant (AA vs. AG/GG) models, to determine odds ratio (OR), 95% confidence intervals (CI) and p-values. These statistical analyses were performed using the PLINK software v.1.07 (http://pngu.mgh.harvard.edu/purcell/plink/) (Purcell et al. 2007).

The association of the rs4420638 polymorphism with obesity-related quantitative traits (e.g., weight, height, BMI, BMI Z-score and waist circumference), by comparing genotypes AA against AG + GG, were tested using the nonparametric Mann-Whitney test. These statistical analyses were performed using the IBM SPSS Statistics software for Windows, version 27 (SPSS, Chicago, IL). A statistically significant association or difference was set at 5%.

## Results

The characteristics of the participants are shown in Table 1 and supplementary Table S1 stratified by sex. The study included a total of 446 children (231 boys and 215 girls): 234 normal-weight and 212 with overweight (including obesity). The obtained genotypes and minor allele frequencies (MAFs) in the total population and the association results with the risk of obesity in a case-control outcome are summarized in Table 2. Minor allele frequency (MAF) for the rs4420638 SNP was 0.146 (G-allele) in the total population, and genotype frequencies were AA 72.87%, AG 25.11% and GG 2.02%. The MAF were 0.145 in boys and 0.147 in girls, and genotype frequencies were AA 73.16%, AG 24.68% and GG 2.16% for boys, and AA 72.56%, AG 25.58% and GG 1.86% for girls (supplementary Table S2). The obtained genotype frequencies were according to the Hardy-Weinberg equilibrium (*p* = 1) in the total population as also in boys and girls (Table 2 and supplementary Table S2).

**Table 1:** Characteristics of the study sample of 446 Portuguese children

Parameters	Total	Normal-weight	OW/OB
N (Males/Females)	446 (231/215)	234	212
Age Range	3.2–13.7	3.2-11.08	4-13.7
Age (years)	7.98 (2.83) 8 (2.83)	7.17 (1.73) 7.33 (2.56)	8.89 (1.95) 8.9 (2.99)
Weight (kg)	32.79 (13.96) 29.27 (18.24)	23.98 (5.2) 23.13 (7.66)	42.51 (12.04) 41.55 (18.75)
Height (cm)	128.91 (12.96) 129.2 (20.03)	122.13 (11.58) 121.9 (16.3)	136.41 (12.48) 137.35 (18.45)
BMI (kg/m^2^)	18.94 (3.91) 17.57 (6.32)	15.89 (1.18) 15.83 (1.58)	22.31 (3.00) 22.20 (4.17)
BMI Z-score	0.919 (1.01) 1 (1.65)	0.12 (0.67) 0.21 (0.98)	1.81 (0.43) 1.83 (0.46)
Waist C (cm)	63.03 (± 10.58) 60 (17.8)	54.96 (4.04) 54.30 (5.13)	71.91 (8.15) 72.00 (10.69)

**Abbreviations**: N, number of individuals; BMI, body mass index; BMI Z-score, body mass index standard deviation score; Waist C, waist circumference; OW/OB, overweight/obesity

Data are presented as mean (SD) (above) and median (IQR) (below) for continuous anthropometric variables

**Table 2:** Association of the rs4420638 polymorphisms with the risk of obesity in the study sample of 446 Portuguese children

Chr:position (hg38)	SNP	Nucleotide change	Genotypes GG/AG/AA	MAF	*P*-HWE	MAF		Model	Association	
						Normal (N = 234)	OW/OB (N = 212)		OR (95% CI)	*p*
19:44919689	rs4420638	c.*459A > G	9/112/325	0.1457	1	0.1731	0.1156	ADD	0.6199 (0.421–0.913)	0.0155
								DOM	0.5875 (0.383-0.9)	0.0145

Abbreviations: N, number of individuals; p-HWE, p-value for Hardy-Weinberg Equilibrium; OR, Odds Ratio; CI, confidence interval; MAF, Minor allele frequency; OW/OB, overweight/obesity; ADD, additive; DOM, dominant

The p-values were obtained by logistic regression under the additive (AA vs. AG vs. GG) and dominant (AA vs. AG/GG) models, comparing children with normal-weight vs. children with overweight and obesity

The frequency of the minor allele was higher in normal-weight individuals in comparison with children with overweight (including obesity), in the total population (0.173 vs. 0.116) as also in boys (0.177 vs. 0.111) and girls (0.169 vs. 0.12). In according to these dissimilar frequencies, logistic regression, comparing children with normal-weight vs. children with overweight and obesity, revealed a significant association with risk of obesity, with the rs4420638 minor G-allele providing protection against obesity in both the additive (OR 0.619; 95% CI 0.421–0.913; *p* = 0.016) and dominant (OR 0.5875; 95% CI 0.383-0.9; *p* = 0.015) models (Table 2). Also, stratifying data by sex, logistic regression revealed near significant protective associations against overweight/obesity for the G-allele, both in boys (OR 0.581; 95% CI 0.321–1.052; *p* = 0.073) and girls (OR 0.595; 95% CI 0.322–1.099; *p* = 0.097) (dominant model) (supplementary Table S2).

The associations between SNP rs4420638 and obesity-related anthropometric traits are detailed in Table 3. Consistently with the case-control results, children homozygous for the major allele (genotype AA) present higher BMI Z-score (mean 0.98) when compared to those carriers of the minor allele (genotypes AG + GG) (mean 0.75) (*p* = 0.049). Moreover, the homozygous individuals for the major allele (genotype AA) revealed significantly higher values for the anthropometric traits weight (*p* = 0.008), height (*p* = 0.023), BMI (*p* = 0.022), and waist circumference (*p* = 0.005), in comparison with carriers of the minor allele (genotypes AG + GG) (Table 3). This same pattern was observed when splitting the sample by sex, although only two significant values were observed, for weight and waist circumference in girls (supplementary Table S3).

**Table 3:** Association of rs4420638 with anthropometric traits in the study sample of 446 Portuguese children

Parameters	Genotype AA	Genotypes AG + GG	*p*
N (Males/Females)	325	112 + 9	-
Age Range	3.3–13.70	3.2–12.7	-
Age (years)	8.08 (1.99) 8 (2.77)	7.35 (2.08) 7.8 (2.89)	0.16
Weight (kg)	33.68 (13.12) 30.3 (19.55)	30.39 (12.36) 26.35 (14.35)	**0.008**
Height (cm)	129.84 (13.86) 129.8 (19.45)	126.4 (13.98) 126.3 (20.20)	**0.023**
BMI (kg/m^2^)	19.18 (3.92) 17.9 (6.43)	18.29 (3.81) 16.92 (5.38)	**0.022**
BMI Z-score	0.98 (0.98) 1.1 (1.62)	0.75 (1.06) 0.82 (1.55)	**0.049**
Waist C (cm)	63.84 (10.67) 61.87 (17.76)	60.86 (10.07) 57.9 (15.38)	**0.005**

Abbreviations: N, number of individuals; BMI, body mass index; BMI Z-score, body mass index standard deviation score; Waist C, waist circumference

Data are presented as mean (SD) (above) and median (IQR) (below) for continuous anthropometric variables. The p-values were obtained using the non-parametric test Mann-Whitney with AG and GG genotypes merged in one group. Significant results (p <0.05) are in bold

## Discussion

In this study, we examined the association of the polymorphism rs4420638 A > G, tagging the LD block harboring the *APOE* and *APOC1* genes, with the risk of obesity using BMI case-control groups of Portuguese children (normal-weight vs. overweight, including obesity). The present study revealed a significant protective effect from the rs4420638 minor G-allele against the risk of obesity in the total population (OR 0.583; 95% CI 0.383-0.9; *p* = 0.015, in the dominant model). Also, near significant protective associations with obesity risk were observed both in boys (*p* = 0.073) and in girls (*p* = 0.097) (dominant model). Moreover, the association with anthropometric variables, by comparing genotype groups (AA vs. AG + GG), revealed the same protective association with lower values for the anthropometric traits of weight, BMI, BMI Z-score, and waist circumference in the carriers of G-allele in the total population. The same pattern was observed in boys and girls when splitting the whole population by sex. These results suggest no sex-specific associations with obesity risk for the rs4420638 variant, in contrast with other genetic loci where sex-dependent effects on adiposity were identified (Jacobsson et al. 2008; Al Asoom et al. 2020).

Numerous association studies have correlated SNP rs4420638 with lipid metabolism by revealing strong associations between the minor G-allele and elevated activity of Lp-PLA2 and high serum levels of TC, TG, LDL cholesterol or HDL cholesterol, thus providing evidence for a significant function in dyslipidemia (Kathiresan et al. 2008; Drenos et al. 2009; Suchindran et al. 2010; Liu et al. 2011; Grallert et al. 2012; Breitling et al. 2015; Zhang et al. 2018; Wang et al. 2022). Curiously, some studies also revealed opposite effects for this locus, showing the rs4420638 A-allele associated with these same high levels of serum lipids (Teslovich et al. 2010; Hoffmann et al. 2018; Bentley et al. 2019; Richardson et al. 2022). The GWAS Catalog EMBL-EBI (http://www.ebi.ac.uk/gwas, accessed on January 24, 2023) (Sollis et al. 2023) provides 156 studies for the variant rs4420638, showing multiple associations with serum lipid traits for both the G and A alleles.

Additionally, while the rs4420638 G-allele was identified conferring protection against obesity (OR 0.48; 95% CI 0.29–0.81; *p* = 0.004) in an Algerian population sample (mean age 44.0) (Boulenouar et al. 2019), a recent study in Chinese children and adolescents (6–18 years old) has not identified rs4420638 as an obesity-linked variant (Wang et al. 2022). Therefore, the observed protective association of the rs4420638 G-allele against obesity risk in Portuguese children is consistent with the study by Boulenouar et al. (2019).

For non-Mendelian diseases, significant protective associations (i.e., the major allele of an associated locus being the risk allele) can be explained by genetic drift, overdominance, frequency-dependent selection, and gene–gene or gene–environment interactions (Kido et al. 2018). However, the mechanism by which the rs4420638A/G variant influences plasma lipid levels and the risk of obesity is unclear. This variant, near the *APOC1* gene, have been reported to exist in LD with APOE ε2/ε3/ε4 polymorphisms (Zhang et al. 2018), and it is unknown whether these associations are independent of the *APOE* alleles that are known to increase the risk of obesity (Galal et al. 2021; Zeljko et al. 2011).

In conclusion, the present study provides further evidence for the *APOE*/*APOC1* candidate-region association with the risk of obesity, in particular in children. Findings revealed the rs4420638 SNP minor G-allele is associated with a lower risk of obesity. This was the first study to describe this significant protective association for the rs4420638 G-allele against obesity risk in childhood exclusively. As the small population sample could be considered a limitation of this study, mainly when stratifying the total population by sex, further investigations with large sample sizes are required to confirm the obtained results.

### Electronic Supplementary Material

Below is the link to the electronic supplementary material.

Supplementary Material 1

## Data Availability

The datasets generated during and/or analysed during the current study are available from the corresponding author on reasonable request.
