# Supplementary material for: Protective Association of APOC1/rs4420638 with Risk of Obesity: A case-control Study in Portuguese Children
Source: Biochem Genet. 2023 Jun 16;62(1):254–63. doi: 10.1007/s10528-023-10427-4 (PMC10902077; doi:10.1007/s10528-023-10427-4)
Supplement: Supplementary file 1 — Supplementary Material 1 [file 10528_2023_10427_MOESM1_ESM.docx]

**Table S1**. Characteristics of the study sample of 446 Portuguese children stratified by sex.

| **Parameters** | **Total** | **Boys** | **Girls** |
| --- | --- | --- | --- |
| N | 446 | 231 | 215 |
| Age Range | 3.2-13.7 | 3.3-12.7 | 3.2-13.7 |
| Age (years) | 7.98 (2.83)  8 (2.83) | 7.89 (2.04)  7.87 (2.68) | 8.09 (2.01)  8.12 (2.99) |
| Weight (kg) | 32.79 (13.96)  29.27 (18.24) | 32.38 (12.28)  29.2 (18.40) | 33.24 (13.73)  29.4 (18.5) |
| Height (cm) | 128.91 (12.96)  129.2 (20.03) | 128.83 (13.95)  129.5 (20.0) | 129.0 (14.01)  128.2 (20.4) |
| BMI (kg/m^2^) | 18.94 (3.91)  17.57 (6.32) | 18.75 (3.67)  17.58 (5.73) | 19.15 (4.16)  17.57 (6.52) |
| BMI Z-score | 0.919 (1.01)  1 (1.65) | 0.89 (0.98)  0.98 (1.68) | 0.94 (1.05)  1.05 (1.57) |
| Waist C (cm) | 63.03 (±10.58)  60 (17.8) | 63.54 (10.27)  61.58 (17.21) | 62.49 (10.9)  59.2 (17.3) |

**Abbreviations**: N, number of individuals; BMI, body mass index; BMI Z-score, body mass index standard deviation score; Waist C, waist circumference; OW/OB, overweight/obesity.

Data are presented as mean (±SD) (above) and median (IQR) (below) for continuous anthropometric variables.

**Table S2.** Association of the rs4420638 polymorphism with the risk of obesity stratified by sex in the study sample of 446 Portuguese children.

| Population | Genotypes  GG/AG/AA | MAF | *P*-HWE | MAF | | Model | Association | |
| --- | --- | --- | --- | --- | --- | --- | --- | --- |
|  |  |  |  | Normal | OW/OB |  | OR (95% CI) | *p* |
| Boys  (N = 231) | 5/57/169 | 0.145 | 1 | 0.177  (N = 119) | 0.111  (N = 112) | ADD | 0.585 (0.342-1.002) | 0.051 |
|  |  |  |  |  |  | DOM | 0.581 (0.321-1.052) | 0.073 |
| Girls  (N = 215) | 4/55/156 | 0.147 | 1 | 0.169  (N = 115) | 0.12  (N = 100) | ADD | 0.660 (0.378-1.154) | 0.145 |
|  |  |  |  |  |  | DOM | 0.595 (0.322-1.099) | 0.097 |

**Abbreviations**: N, number of individuals; *p*-HWE, *p*-value for Hardy-Weinberg Equilibrium; OR, Odds Ratio; CI, confidence interval; MAF, Minor allele frequency; OW/OB, overweight/obesity; ADD, additive; DOM, dominant.

The p-values were obtained by logistic regression under the additive (AA vs. AG vs. GG) and dominant (AA vs. AG/GG) models, comparing children with normal-weight vs. children with overweight and obesity. Significant results (*p* <0.05) are in bold

**Table S3**. Association of rs4420638 with anthropometric traits stratified by sex in the study sample of 446 Portuguese children (231 boys and 215 girls).

|  | Boys | | | Girls | | |
| --- | --- | --- | --- | --- | --- | --- |
| Parameters | Genotype  AA | Genotypes  AG + GG | *P* | Genotype  AA | Genotypes  AG + GG | *p* |
| N | 169 | 57 + 5 | - | 156 | 55 + 4 | - |
| Age Range | 3.3-12.3 | 3.45-12.7 | - | 3.48-13.7 | 3.2-11.7 | - |
| Age (years) | 7.97 (1.98)  7.9 (2.57) | 7.68 (2.19)  7.75 (3.05) | 0.364 | 8.2 (2.01)  8.32 (3.01) | 7.8 (1.98)  8 (2.65) | 0.265 |
| Weight (kg) | 33.07 (12.16)  29.75 (19.38) | 30.5 (12.55)  27.95 (15.76) | 0.089 | 34.35 (14.12)  30.75 (20.02) | 30.29 (12.27)  26.3 (13.85) | **0.041** |
| Height (cm) | 129.76 (13.87)  130 (19.25) | 126.31 (13.98)  126 (22.05) | 0.113 | 129.95 (13.91)  129.3 (20.25) | 126.51 (14.11)  127 (20.4) | 0.103 |
| BMI (kg/m^2^) | 18.89 (3.52)  17.8 (5.96) | 18.38 (4.07)  16.94 (5.75) | 0.137 | 19.51 (4.32)  18.16 (7.04) | 18.22 (3.57)  16.84 (5.21) | 0.068 |
| BMI Z-score | 0.98 (0.93)  1.02 (1.65) | 0.73 (1.12)  0.79 (1.69) | 0.215 | 1 (1.05)  1.2 (1.56) | 0.78 (1.02)  0.87 (1.43) | 0.094 |
| Waist C (cm) | 64.14 (10.21)  62.18 (18.64) | 61.93 (10.34)  59.08 (18.64) | 0.105 | 63.53 (11.17)  60.2 (18.14) | 59.75 (9.76)  57 (13.6) | **0.021** |

**Abbreviations**: N, number of individuals; BMI, body mass index; BMI Z-score, body mass index standard deviation score; Waist C, waist circumference;

Data are presented as mean (±SD) (above) and median (IQR) (below) for continuous anthropometric variables. The p-values were obtained using the non-parametric test Mann-Whitney with AG and GG genotypes merged in one group. Significant results (*p* <0.05) are in bold.
